# Supplementary material for: Drying of the Natural Fibers as A Solvent-Free Way to Improve the Cellulose-Filled Polymer Composite Performance
Source: Polymers (Basel). 2020 Feb 21;12(2):484. doi: 10.3390/polym12020484 (PMC7077673; doi:10.3390/polym12020484)
Supplement: Supplementary file 1 [file polymers-12-00484-s001.pdf]

polymers

Drying of the Natural Fibers as A Aolvent-Free Way to Improve the Cellulose-Filled Polymer Composite Performance

Stefan Cichosz [1] and Anna Masek [1,*]

[1] Institute of Polymer and Dye Technology, Faculty of Chemistry, Lodz University of Technology, Stefanowskiego 12/16, 90-924 Lodz, Poland
* Correspondence: anna.masek@p.lodz.pl

1. Cellulose moisture content investigation

1.1. Near infrared spectroscopy (NIR)

1.1.1. Method parameters

In this method, the near-infrared region of the electromagnetic spectrum was used. The measurements were carried out in the range of 10000-4000 cm^{-1} in adsorption mode—64 scans (Thermo Scientific, Nicolet 6700). Cellulose fibres were dried for 24 h at 100 °C (Binder® oven; crystallizer 70x40 mm) before being analysed.

1.1.2. NIR Characterization

The processes of water adsorption and desorption has been also tracked with the employment of NIR technique which, according to literure, is more fragile to polar groups [1–3]. Therefore, it seems as a perfect tool in the cellulose moisture content investigation [4].

Table 1. Tabularized absorption bands assigned to the chemical groups.

Wavenumber [cm^{-1}]	*Chemical group*	*Ref.*
4750	-OH, C=O	[5]
5220-5150	-OH, water	[6]
6709	-OH, water, hydrogen bonds	[7]
6772	-OH	[8]
8165	C-H	[6]

Considering the NIR spectra (**Figure 1** and **Figure 2**) of cellulose fibres some signals characteristic of this substance can be recognized, e.g., 8165 cm^{-1} (C-H stretching of the second overtone) [6], 7290 cm^{-1} (C-H stretching and C-H deformation vibrations) [6], 6472 cm^{-1} (O-H stretching of the first overtone) [6], 4754 cm^{-1} (O-H stretching vibrations) [5]. Absorption bands assigned to chemical groups are tabularized in **Table 1**.

Considering the data gathered in **Figure 1**, which describes the changes in the water content during the moisture adsorption process, some variations are visible. Firstly, the intensity increases at 6709 cm^{-1} and 5174 cm^{-1} are revealed. These signals are assigned to the adsorbed moisture [6,7]. Nevertheless, different absorption bands are also influenced by the changes in cellulose water uptake, e.g., 4751 cm^{-1} (-OH moieties) [5].

Furthermore, regarding **Figure 2** describing the moisture desorption process, also some changes in the intensities of 6709 cm^{-1} and 5174 cm^{-1} might be noticed. Nevertheless, differences observed at 6709 cm^{-1} signal are slighter than the ones visible in Figure 15. This may indicate some information about the diversified rates of water adsorption and desorption processes, as NIR technique is more fragile to changes in moisture content [9]. On the other hand, observed results might be the due to the water evolution of different states [10–12].

Moreover, considering data gathered in **Figure 1** and **Figure 2**, the water content is possible to be tracked with 5174 cm^{-1} absorption band as the intensity changes between the analysed fibres are possible to be distinguished. Furthermore, contrary to data obtained with the FT-IR, according to presented NIR spectra, it may be said that the water desorption process rate is faster at the beginning. The confirmation of this phenomenon could be the fact that intensity at 5174 cm^{-1} drops significantly after 45 min of cellulose drying (**Figure 2**). However, regarding the same signal in **Figure 1**, it is clearly seen that the intensity increase caused by the water adsorption seems to be steadier and slower.

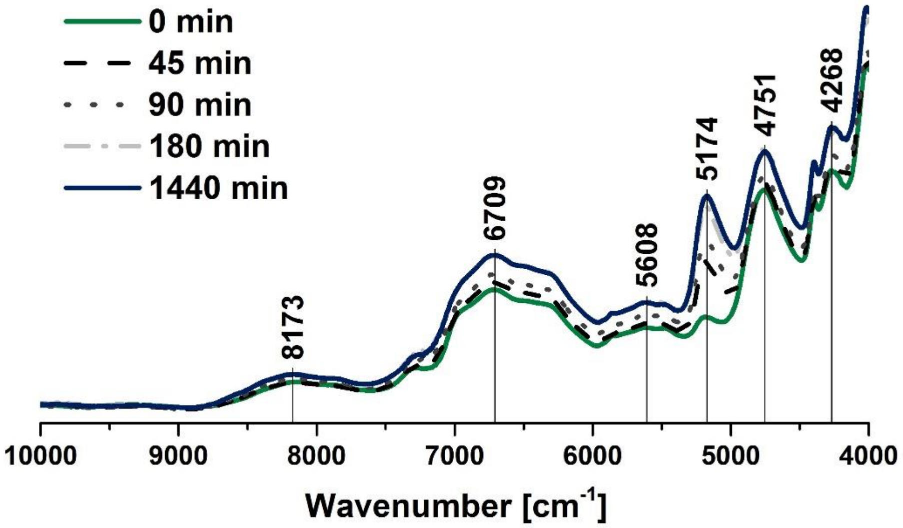

Figure 1. NIR spectra of cellulose fibres dried for 1440 min at 100 °C and then left for moisture adsorption for different times. Characteristic absorption bands: 8165 cm^{-1} (C-H), 7290 cm^{-1} (C-H), 6772 cm^{-1} (O-H), 6706 cm^{-1} (O-H, water), 4754 cm^{-1} (O-H).

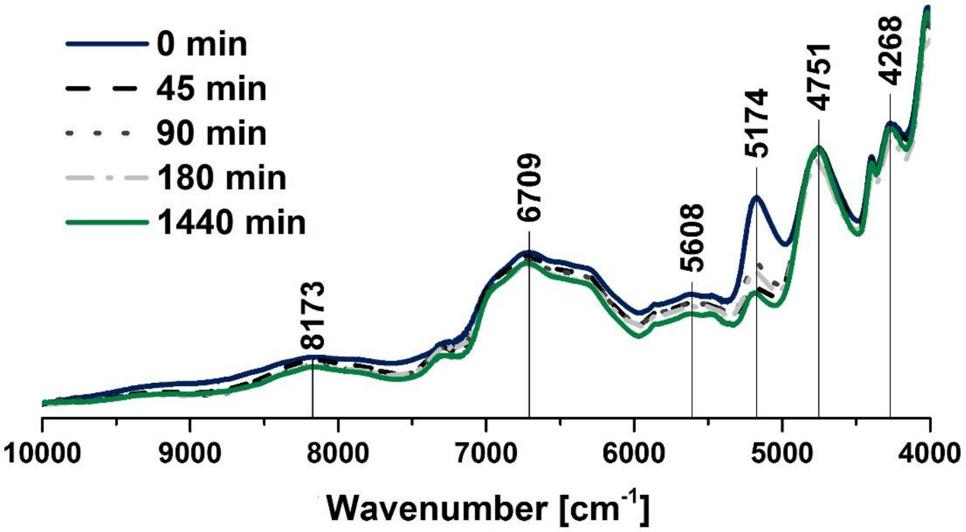

Figure 2. NIR spectra of cellulose fibres dried for 45, 90, 180, 1440 min at 100 °C. Characteristic absorption bands: 8165 cm^{-1} (C-H), 7290 cm^{-1} (C-H), 6772 cm^{-1} (O-H), 6706 cm^{-1} (O-H, water), 4754 cm^{-1} (O-H).

1.2. Differential scanning calorimetry (DSC)

1.2.1. Experiment conditions

Cellulose fibres were dried for 24 h at 100 °C (Binder® oven; crystallizer 70 × 40 mm) before being analysed. Differential scanning calorimetry (DSC) investigation has been performed in a

temperature range from -20–200 °C (heating rate: 10 °C/min; Ar 60 cm³/min) prior to analyse the water evaporation process establishing its enthalpy (ΔH) and temperature of the peak (T_{peak}). Here, as well, Mettler Toledo TGA/DSC 1 STARe System equipped with Gas Controller GC10 has been employed.

1.2.2. DSC analysis

According to the performed differential scanning calorimetry analysis, the water evaporation process may be observed. The endothermic peak visible in **Figure 3** is assigned to the moisture desorption from the surface of the analysed biopolymer [13,14].

What should be underlined, the significant difference in the sample thermal behaviour between the dried and not dried cellulose may be detected. Considering UFC100/ND, a crucial increase in the enthalpy assigned to the water evaporation might be noticed, while in case of UFC100/D/1440 the endothermic peak is only slight. This proves the good efficiency of thermal drying.

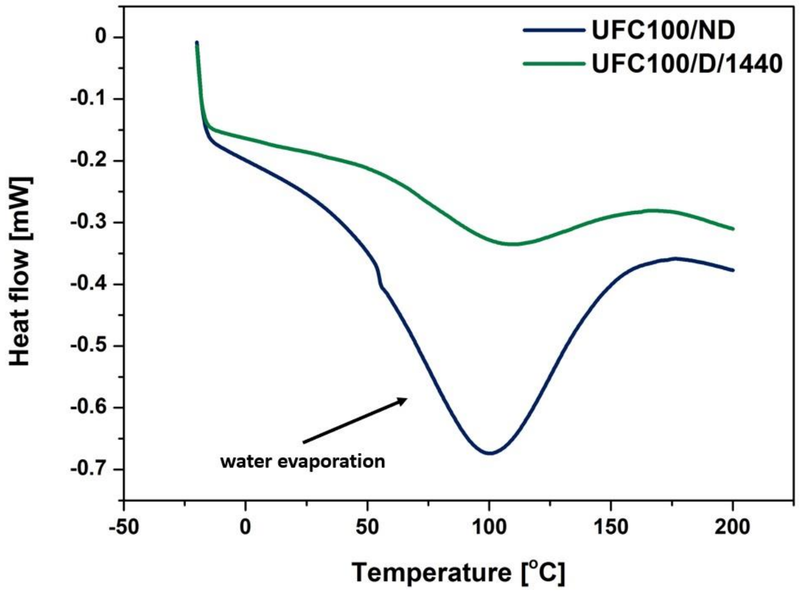

Figure 3. Comparison of dried and not dried cellulose water evaporation phenomenon according to the DSC curves.

Moreover, according to the data given in **Table 2**, the difference in the enthalpy value is also significant. ΔH in case of UFC100/ND specimen is approximately 4 times higher in comparison with UFC100/D/1440. Elevated enthalpy change value means more energy required to desorb the water from the cellulose fibre surface as dehydration heat increases considerably when the sample exhibits raised moisture level [15]. What is also interesting, the T_{peak} of water evaporation shifts from 98°C (UFC100/ND) to 105 °C has been detected (UFC100/D/1440). This could be due to some structural changes caused by the thermal treatment [16,17].

Table 2. Tabularized water evaporation enthalpy values for died cellulose samples.

Sample	T_{peak} [°C]	ΔH [J/g]
UFC100/ND	98	175.84
UFC100/D/1440	105	41.09

2. Polymer composite samples investigation

2.1. Thermogravimetric analysis (TGA)

2.1.1. Method parameters

Thermogravimetric analysis (TGA) has been used in order to get acquainted with thermal degradation process detecting the mass loss as a function of raising temperature in the range from 25–600 °C (heating rate: 10 °C/min; air 50 cm³/min). Mettler Toledo TGA/DSC 1 STARe System equipped with Gas Controller GC10 has been employed in this investigation. Activation energy (E_A) values for the following decomposition steps are calculated with the use of Broido's methos [18]:

$$y = \frac{m_t - m_\infty}{m_0 - m_\infty}$$

$$\ln\left[\ln\left(\frac{1}{y}\right)\right] = -\frac{E_A}{R}\cdot\frac{1}{T} + C \quad as \quad a \quad linear \quad function: Y = aX + b$$

$$where: Y = \ln\left[\ln\left(\frac{1}{y}\right)\right], X = \frac{1}{T}, a = -\frac{E_A}{R}, b = C$$

$$therefore \quad E_A = -a \cdot R$$

where:

m_t – specimen mass at the time *t* [g]

m_0 – specimen mass at the beginning of considered decomposition step [g]

m_∞ – specimen mass at the end of considered decomposition step [g]

T – temperature [K]

R – gas constant [8.31 J/(mol·K)]

2.1.2. TGA investigation

Thermogravimetric analysis has been employed in order to investigate the thermal decomposition of analysed polymer composites. In the **Figure 4**, TGA curves which are typical for ethylene-norbornene copolymer filled with cellulose fibres might be observed. It consists of two significant decomposition steps. First one is considered to be connected with the biopolymer thermal degradation and the second one—assigned to the polymer matrix disintegration. Moreover, also a TGA curve of neat polymer matrix (TOPAS) is presented. In this case only one decomposition step around 350 °C is evidenced.

According to the data given in Table 14, it may be observed that filled systems exhibit lower $T_{05\%}$, which is considered to be the initial decomposition temperature, in comparison with the neat polymer matrix. Furthermore, it is clearly visible that the most dynamic mass loss of TOPAS is detected from 350–500°C. It proves higher thermal resistance of neat polymer matrix while comparing with the other analysed composite samples.

On the other hand, in case of filled systems, the most dynamic mass loss for investigated composite samples is detected between 300 °C and 500 °C. This phenomenon has been reported before in literature [19]. Furthermore, around 360 °C cellulose reveals a significant drop (mass loss of almost 80%) [20]. Nevertheless, all investigated samples of cellulose-filled TOPAS, according to data gathered in **Table 3** and **Figure 4**, follow the same degradation path and the differences between the specimens are not relevant. Furthermore, it may be noticed that from approximately 450 °C on, all analysed samples begin to degrade in the same rate. This is the moment that cellulose fibres are fully degraded.

Table 3. Temperatures of the mass loss; $T_{x\%}$ - temperature at which the mass loss of x% is detected.

Sample	***$T_{05\%}$ [°C]***	***$T_{10\%}$ [°C]***	***$T_{15\%}$ [°C]***	***$T_{20\%}$ [°C]***	***$T_{50\%}$ [°C]***	***$T_{80\%}$ [°C]***	***$T_{90\%}$ [°C]***
TOPAS	411	430	440	447	468	479	483
TOPAS + UFC100/ND	333	345	377	419	459	474	479
TOPAS + UFC100/D/45	333	344	372	411	457	474	480

TOPAS + UFC100/D/180	331	343	366	403	453	471	477
TOPAS + UFC100/D/1440	331	343	366	405	456	473	478

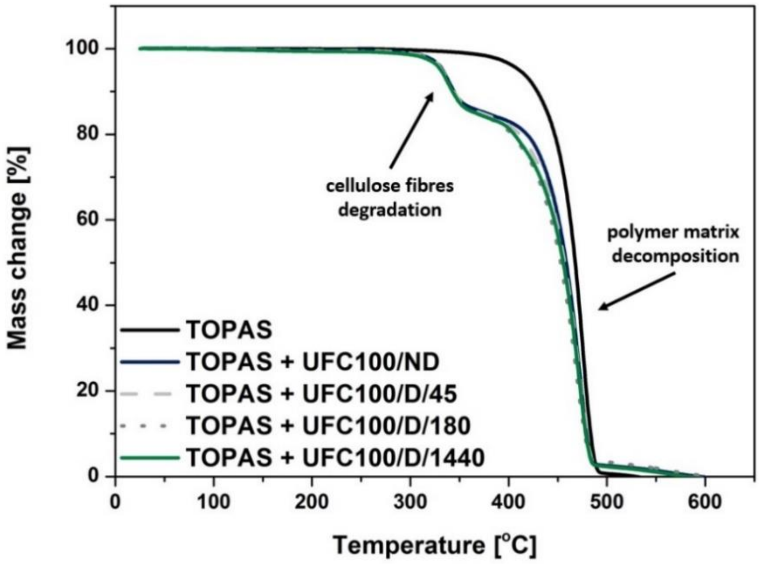

Figure 4. TGA curves of analysed composites filled with cellulose fibres dried for different times.

Moreover, **Table 4** reveals activation energies assigned to cellulose degradation (E_{A1}) and polymer matrix decomposition (E_{A_2}) which have been calculated according to the Broido's approach [18]. It may be observed again that drying has almost no impact on the degradation behaviour of analysed composite samples. Moreover, what should be underlined, the activation energy of neat TOPAS degradation is comparable with the activation energy values presented for different composites. Therefore, it may be stated that the degradation of polymer matrix itself is not affected by the presence of cellulose fibres and the reason for lower cellulose-filled polymer composites thermal stability is the presence of biopolymer. Decreased thermal resistance of cellulose-filled samples has been also reported in other research study [21]. Moreover, this could be explained by the lower thermal stability of cellulose in comparison with the polymer matrix [22] which is a consequence of -OH groups presence [23].

Table 4. Tabularized values of activation energy assigned to the decomposition steps calculated with the use of Broido's method [18]; E_{A_1}—activation energy of cellulose degradation, E_{A_2}–activation energy of polymer matrix decomposition.

Sample	E_{A_1} *[kJ/mol]*	E_{A_2} *[kJ/mol]*
TOPAS	-	$\mathbf{225 \pm 3}$
TOPAS + UFC100/ND	$\mathbf{201 \pm 3}$	$\mathbf{241 \pm 2}$
TOPAS + UFC100/D/45	$\mathbf{195 \pm 2}$	$\mathbf{236 \pm 2}$
TOPAS + UFC100/D/180	$\mathbf{201 \pm 2}$	$\mathbf{228 \pm 2}$
TOPAS + UFC100/D/1440	$\mathbf{189 \pm 2}$	$\mathbf{232 \pm 2}$

2.2. Differential scanning calorimetry (DSC)

2.2.1. Experiment parameters

Differential scanning calorimetry (DSC) investigation has been performed in a temperature range from −40–200°C (heating rate: 10 °C/min; argon atmosphere) prior to analyse changes in glass

transition temperature of ethylene elastic segments (Tg_1), glass transition temperature of rigid norbornene segments (Tg_2) and softening enthalpy (ΔH). Here, as well, Mettler Toledo TGA/DSC 1 STARe System equipped with Gas Controller GC10 has been employed.

2.2.2. DSC analysis

Differential scanning calorimetry (DSC) was used in order to assess the filler influence on the glass transition temperature process of ethylene (Tg_1) and norbornene (Tg_2) segments, as well as, the phenomenon of material softening and its enthalpy change value (ΔH). In **Figure 5** DSC curves of composites filled with neat cellulose fibres dried for different times are presented. Firstly, one may observe that the curve shapes are similar to each other. However, some differences in softening enthalpy value and shifts in glass transition temperatures between neat polymer matrix and filled TOPAS are detected.

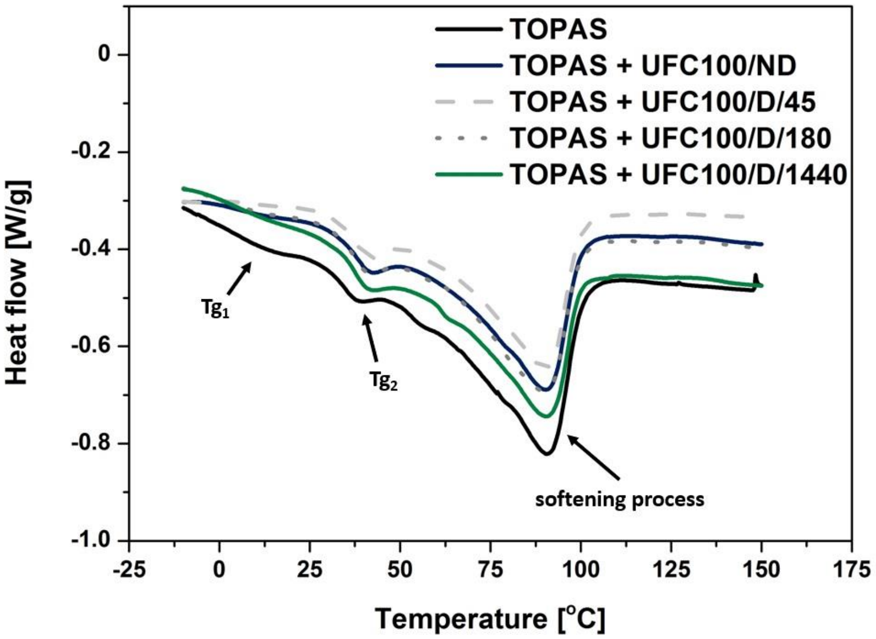

Figure 5. DSC curves of analysed composites filled with cellulose fibres dried for different times: Tg_1 —glass transition of ethylene segments, Tg_2—glass transition of norbornene rings segments.

Giving a closer look on data gathered in **Table 5**, it may be observed that there are some major differences between the neat polymer matrix and cellulose-filled TOPAS, namely, Tg_1 fall from 9°C (TOPAS) to approximately 3.5°C (filled composite), enthalpy change from 54 J/g (TOPAS) to roughly 41 J/g (filled composite). Yet, the impact of UFC100 incorporation on norbornene segments glass transition temperature (Tg_2) is not relevant and there are no variations considering temperature of this transition (T_{peak}). This phenomenon has been observed before in different studies[24–26]. Moreover, described above decrease in softening enthalpy (ΔH) also has been reported before[27,28].

Table 5. Tabularized maximum values of ethylene segments glass transition temperatures (Tg_1), norbornene segments glass transition temperatures (Tg_2), peak temperature (T_{peak}) of the softening process and its enthalpy change (ΔH).

Sample	*Tg_1 [°C]*	*Tg_2 [°C]*	*T_{peak} [°C]*	*ΔH [J/g]*
TOPAS	9	36	90	53.76
TOPAS + UFC100/ND	3	39	91	41.45
TOPAS + UFC100/D/45	4	39	90	41.37

TOPAS + UFC100/D/180	3	38	90	41.88
TOPAS + UFC100/D/1440	4	38	90	40.09

Nonetheless, what should be undoubtedly underlined, filled samples does not vary between each other. Parameters established via DSC analysis are at the same level in case of all cellulose-filled samples and the drying time has no influence on them.

2.3. Surface free energy (SFE)

2.3.1. Method Explanation

Surface free energy has been determined on the basis of contact angle measurements done for three liquids: distilled water, ethylene glycol, 1,4-diiodomethane. Droplets had a volume of approximately 2 μL. Surface of polymer composites has been cleaned with the use of acetone before the contact angle measurements was done. OCA 15EC goniometer by DataPhysics Instruments GmbH® equipped with single direct dosing system (0.01-1 mL B. Braun® syringe) was employed. Surface free energy is calculated thanks to the Owens–Wendt–Rabel–Kaelble (OWRK) method [29]:

$$E = E_P + E_D$$

$$\frac{\sigma_L(1+\cos\Theta)}{2\sqrt{\sigma_L^D}} = \sqrt{\sigma_S^P}\cdot\sqrt{\frac{\sigma_L^P}{\sigma_L^D}} + \sqrt{\sigma_S^D} \quad as\ \ a\ \ linear\ \ function: Y = a\cdot X + b$$

$$while:\ \ Y = \frac{\sigma_L(1+\cos\Theta)}{2\sqrt{\sigma_L^D}}, X = \sqrt{\frac{\sigma_L^P}{\sigma_L^D}}, a = \sqrt{\sigma_S^P}, b = \sqrt{\sigma_S^D}$$

$$therefore\ \ E_P = a^2 = \sigma_S^P\ \ and\ \ E_D = b^2 = \sigma_S^D$$

where:

E – total surface free energy [mJ/m^2]

E_P – polar part of surface free energy [mJ/m^2]

E_D – dispersive part of surface free energy [mJ/m^2]

σ_L – total liquid surface tension [mN/m]

σ_L^P, σ_L^D – respectively: polar and dispersive part of liquid surface tension [mN/m]

σ_S^P, σ_S^D – respectively: polar and dispersive part of solid surface tension [mN/m]

Θ – contact angle [∘]

2.3.2. SFE investigation

Due to the cellulose drying time, some variations of composite samples surface free energy have been detected. Similar changes have been also reported in literature [30]. Regarding **Figure 6**, neat polymer matrix exhibit the surface free energy which is approximately E = (40 ± 1) mJ/m^2. Consequently, it may be claimed that E drops significantly while TOPAS is loaded with cellulose fibres. All polymer composites filled with natural fibres dried for different times exhibit a surface free energy of almost 30 mJ/m^2.

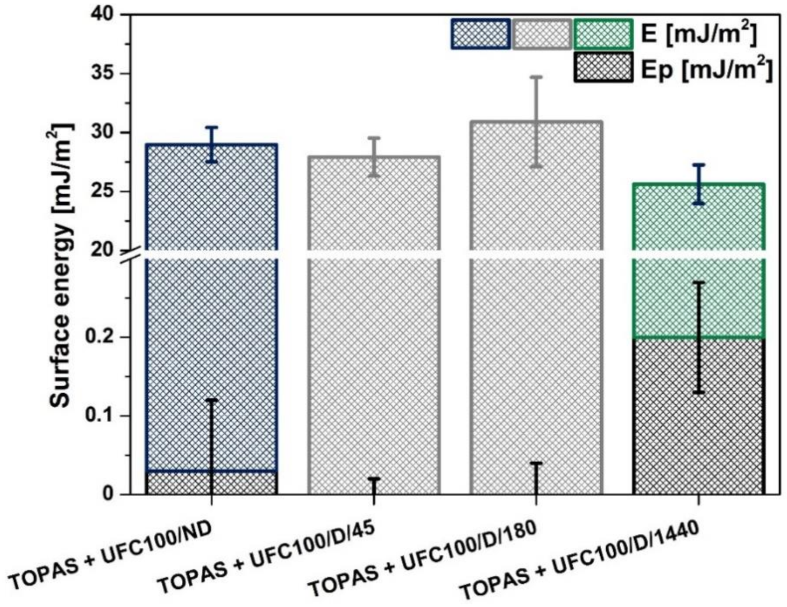

Figure 6. Surface free energy of investigated composites filled with modified cellulose fibres: a) not dried before the modification process, b) dried before the modification process.

Moreover, the polar part of surface free energy, in case of unfilled TOPAS, is (0.10 ± 0.06) mJ/m^2. E_p values obtained in case of the composites filled with not dried cellulose fibres, either dried for 45 min and 180 min, are lower than the surface free energy polar part assigned to neat polymer matrix. However, TOPAS + UFC100/D/1440 specimen exhibits the elevated polar part of surface free energy value of approximately 0.2 mJ/m^2.

Described above changes may be important considering the wetting of prepared composite materials, e.g., the lower the surface energy is, the higher contact angle value in case of water [31]. What is more, the polar and dispersive components of surface free energy are also crucial in case of wetting [32]. Due to their ratio, prepared composite would be more or less fragile to polar either non-polar solvents [33].

References

1. Ambjörnsson, H.A.; Schenzel, K.; Germgård, U. Carboxymethyl cellulose produced at different mercerization conditions and characterized by nir ft raman spectroscopy in combination with multivariate analytical methods. *BioResources* **2013**, *8*, 1918–1932.
2. Schenzel, A.; Hufendiek, A.; Barner-Kowollik, C.; Meier, M.A.R. Catalytic transesterification of cellulose in ionic liquids: Sustainable access to cellulose esters. *Green Chem.* **2014**, *16*, 3266–3271.
3. He, W.; Hu, H. Prediction of hot-water-soluble extractive, pentosan and cellulose content of various wood species using FT-NIR spectroscopy. *Bioresour. Technol.* **2013**, *140*, 299–305.
4. Delwiche, S.R.; Pitt, R.E.; Norris, K.H. Examination of Starch-Water and Cellulose-Water Interactions With Near Infrared (NIR) Diffuse Reflectance Spectroscopy. *Starch - Stärke* **1991**, *43*, 415–422.
5. Ali, M.; Emsley, A.M.; Herman, H.; Heywood, R.J. Spectroscopic studies of the ageing of cellulosic paper. *Polymer (Guildf).* **2001**, *42*, 2893–2900.
6. Schwanninger, M.; Rodrigues, J.C.; Fackler, K. A review of band assignments in near infrared spectra of wood and wood components. *J. Near Infrared Spectrosc.* **2011**, *19*, 287–308.
7. Inagaki, T.; Siesler, H.W.; Mitsui, K.; Tsuchikawa, S. Difference of the crystal structure of cellulose in wood after hydrothermal and aging degradation: A NIR spectroscopy and XRD study. *Biomacromolecules* **2010**, *11*, 2300–2305.
8. Rantanen, J.; Räsänen, E.; Tenhunen, J.; Känsäkoski, M.; Mannermaa, J.P.; Yliruusi, J. In-line moisture measurement during granulation with a four-wavelength near infrared sensor: An evaluation of particle size and binder effects. *Eur. J. Pharm. Biopharm.* **2000**, *50*, 271–276.
9. Greensill, C. V.; Walsh, K.B. Optimization of instrumentation precision and wavelength resolution for the performance of NIR calibrations of sucrose in a water-cellulose matrix. *Appl. Spectrosc.* **2000**, *54*, 426–430.

10. Scheirs, J.; Camino, G.; Tumiatti, W. Overview of water evolution during the thermal degradation of cellulose. *Eur. Polym. J.* **2001**, *37*, 933–942.
11. Hay, J.N.; Laity, P.R. Observations of water migration during thermoporometry studies of cellulose films. *Polymer (Guildf).* **2000**, *41*, 6171–6180.
12. Hatakeyama, T.; Tanaka, M.; Hatakeyama, H. Thermal properties of freezing bound water restrained by polysaccharides. *J. Biomater. Sci. Polym. Ed.* **2010**, *21*, 1865–1875.
13. Jandura, P.; Riedl, B.; Kokta, B. V. Thermal degradation behavior of cellulose fibers partially esterified with some long chain organic acids. *Polym. Degrad. Stab.* **2000**, *70*, 387–394.
14. Tsujiyama, S.I.; Miyamori, A. Assignment of DSC thermograms of wood and its components. *Thermochim. Acta* **2000**, *351*, 177–181.
15. Ciolacu, D.; Ciolacu, F.; Popa, V.I. Amorphous cellulose – structure and characterization. *Cellul. Chem. Technol* **2011**, *45*, 13–21.
16. Luo, X.L.; Zhu, J.Y.; Gleisner, R.; Zhan, H.Y. Effects of wet-pressing-induced fiber hornification on enzymatic saccharification of lignocelluloses. *Cellulose* **2011**, *18*, 1055–1062.
17. Salam, A.; Lucia, L.A.; Jameel, H. A novel cellulose nanocrystals-based approach to improve the mechanical properties of recycled paper. *ACS Sustain. Chem. Eng.* **2013**, *1*, 1584–1592.
18. Broido, A. A simple, sensitive graphical method of treating thermogravimetric analysis data. *J. Polym. Sci. Part A-2 Polym. Phys.* **1969**, *7*, 1761–1773.
19. Perisić, M.; Radojević, V.; Uskoković, P.S.; Stojanović, D.; Jokić, B.; Aleksić, R. Wood-thermoplastic composites based on industrial waste and virgin high-density polyethylene (HDPE). *Mater. Manuf. Process.* **2009**, *24*, 1207–1213.
20. Müller-Hagedorn, M.; Bockhorn, H.; Krebs, L.; Müller, U. A comparative kinetic study on the pyrolysis of three different wood species. *J. Anal. Appl. Pyrolysis* **2003**, *68–69*, 231–249.
21. Singh, S.; Mohanty, A.K. Wood fiber reinforced bacterial bioplastic composites: Fabrication and performance evaluation. *Compos. Sci. Technol.* **2007**, *67*, 1753–1763.
22. Ashori, A.; Nourbakhsh, A. Composites : Part B Performance properties of microcrystalline cellulose as a reinforcing agent in wood plastic composites. *Compos. Part B* **2010**, *41*, 578–581.
23. Fernandes, E.G.; Pietrini, M.; Chiellini, E. Bio-Based Polymeric Composites Comprising Wood Flour as Filler. **2004**, *7*, 1200–1205.
24. Cichosz, S.; Masek, A.; Wolski, K. Innovative cellulose fibres reinforced ethylene-norbornene copolymer composites of an increased degradation potential. *Polym. Degrad. Stab.* **2019**, *159*, 174–183.
25. Pasquini, D.; Teixeira, E. de M.; Curvelo, A.A. da S.; Belgacem, M.N.; Dufresne, A. Surface esterification of cellulose fibres: Processing and characterisation of low-density polyethylene/cellulose fibres composites. *Compos. Sci. Technol.* **2008**, *68*, 193–201.
26. Lodha, P.; Netravali, A.N. Thermal and mechanical properties of environment-friendly "green" plastics from stearic acid modified-soy protein isolate. *Ind. Crops Prod.* **2005**, *21*, 49–64.
27. Freire, C.S.R.; Silvestre, A.J.D.; Neto, C.P.; Gandini, A.; Martin, L.; Mondragon, I. Composites based on acylated cellulose fibers and low-density polyethylene: Effect of the fiber content, degree of substitution and fatty acid chain length on final properties. *Compos. Sci. Technol.* **2008**, *68*, 3358–3364.
28. Marcovich, N.E.; Villar, M.A. Thermal and mechanical characterization of linear low-density polyethylene/wood flour composites. *J. Appl. Polym. Sci.* **2003**, *90*, 2775–2784.
29. Encinas, N.; Pantoja, M.; Abenojar, J.; Martínez, M.A. Control of wettability of polymers by surface roughness modification. *J. Adhes. Sci. Technol.* **2010**, *24*, 1869–1883.
30. Rudawska, A.; Jakubowska, P.; Kloziński, A. Surface free energy of composite materials with high calcium carbonate filler content. **2017**, 434–440.
31. Coulson, S.R.; Woodward, I.; Badyal, J.P.S.; Brewer, S.A.; Willis, C.; Down, P.; Ojq, S.S.P. Super-Repellent Composite Fluoropolymer Surfaces. **2000**, 8836–8840.
32. Arbelaiz, A.; Fernández, B.; Ramos, J.A.; Retegi, A.; Llano-Ponte, R.; Mondragon, I. Mechanical properties of short flax fibre bundle/polypropylene composites: Influence of matrix/fibre modification, fibre content, water uptake and recycling. *Compos. Sci. Technol.* **2005**, *65*, 1582–1592.
33. Aranberri-Askargorta, I.; Lampke, T.; Bismarck, A. Wetting behavior of flax fibers as reinforcement for polypropylene. **2003**, *263*, 580–589.
